# Strategic Actions to Value, Conserve, and Restore the Natural Capital of Megadiversity Countries: The Case of Mexico

**DOI:** 10.1093/biosci/biu195

**Published:** 2014-12-12

**Authors:** José Sarukhán, Tania Urquiza-Haas, Patricia Koleff, Julia Carabias, Rodolfo Dirzo, Exequiel Ezcurra, Sergio Cerdeira-Estrada, Jorge Soberón

**Affiliations:** José Sarukhán is the national coordinator of the National Commission for Knowledge and Use of Biodiversity (CONABIO), in Mexico City, Mexico. Tania Urquiza-Haas (turquiza@conabio.gob.mx) is the coordinator of ecosystem assessments at CONABIO. Patricia Koleff is the director of analysis and priorities at CONABIO. Julia Carabias is a professor in the School of Sciences at the National Autonomous University of Mexico, in Mexico City. Rodolfo Dirzo is a professor in the Department of Biology at Stanford University, in Stanford, California. Exequiel Ezcurra is a professor at the University of California and director of the Institute for Mexico and the United States, in Riverside. Sergio Cerdeira-Estrada is the coordinator of marine monitoring at CONABIO. Jorge Soberón is a professor in the Department of Ecology and Evolutionary Biology and a senior scientist at the Biodiversity Institute at Kansas University, in Lawrence.

**Keywords:** ecosystem assessment, science–policy gaps, conservation instruments, sustainable resource use, environmental stewardship

## Abstract

Decisionmakers need updated, scientifically sound and relevant information to implement appropriate policy measures and make innovative commitments to halt biodiversity loss and improve human well-being. Here, we present a recent science-based synthesis on the biodiversity and ecosystem services of Mexico, intended to be a tool for policymakers. We describe the methodological approach used to undertake such an assessment and highlight the major findings. Organized into five volumes and originally written in Spanish (Capital Natural de México), it summarizes the available knowledge on the components, structure, and functioning of the biodiversity of Mexico; the threats and trajectories of anthropogenic impact, together with its conservation status; and the policies, institutions, and instruments available for its sustainable management. We stress the lessons learned that can be useful for similar exercises in other megadiverse developing countries and identify major gaps and strategic actions to conserve the natural capital in light of the challenges of the Anthropocene.

**Four decades have passed since the first call for** action on the global environmental crisis by the international community (United Nations Conference on the Human Environment, Stockholm, 1972). Over the last 40 years or so, numerous scientific studies have drawn our attention to the magnitude and range of consequences of human impacts on biodiversity and ecosystem processes (see Barnosky et al. 2014) and have alerted us that population growth and aggregate, unequally distributed consumption cannot continue unchecked on a finite planet (Ehrlich and Ehrlich 2013). New terms and concepts have also emerged to increase knowledge of the impact of human activities as a planetary force in its own right, such as *Anthropocene* (Crutzen et al. 2002) and *planetary boundaries*, which emphasizes the natural limitations that constrain human activities to a safe operating space (Rockström et al. 2009).

Despite our growing understanding of the problems involved and conservation efforts supported by governments and millions of people worldwide (Rands et al. 2010), global development models and decisionmaking criteria about resource management have failed to keep up with the anthropogenic thrust of change (Stafford-Smith et al. 2012). At the heart of the problem lies a disconnect, at many scales, between what scientists know about the functioning of ecosystems and the operation of the economy, including the structures of subsidies, market failures, distorted policies, and decisions based on private interests and a short-term vision (Wood et al. 2000).

At the global scale, a large volume of documents communicating the fundamental importance of biodiversity for human well-being have been generated by the Millennium Ecosystem Assessment (MA 2005) and the Economics of Ecosystems and Biodiversity initiative (TEEB 2008). The Millennium Ecosystem Assessment was particularly important, because it stimulated the synthesis and evaluation of our knowledge about the links between human well-being and ecosystems in a policy-relevant manner and encouraged a set of responses to manage ecosystems in a sustainable way. The Economics of Ecosystems and Biodiversity initiative highlighted the growing economic costs represented by biodiversity loss and ecosystem degradation.

Although global assessments are not, by definition, intended to address local problems (the level at which most decisions affecting land use change and ecosystem loss or degradation take place), they provide guidance to influence policies and their implementation at national scales and, combined with finer-scale research, can help to focus on environmental problems at different levels of governance (Soberón and Sarukhán 2010, DeFries et al. 2012).

National assessments are the next natural step in connecting science and policymakers. In 2005, a major effort of this type was launched in Mexico. This was a country-level assessment of the state of knowledge, the status of the components, and the function of biodiversity, and approaches to its conservation and management. The intended audience was the academy; nongovernmental organizations; and the government, mostly at a federal level. The effort was started for two reasons: First, despite substantial achievements, the pace of ecosystem degradation and biodiversity loss is still unacceptably high in Mexico. It was considered indispensable to obtain updated figures in order to plan improved conservation actions. Second, in 2005, the Mexican National Commission on Biodiversity (CONABIO) had already compiled a wealth of data and information about the components and structure of the biodiversity of Mexico. Therefore, summarizing it to make it widely available to large sectors of society and organizing it for the purposes of guiding policy became the central objectives of the effort. In this article, we discuss the experience of undertaking Mexico's ecosystem assessment and highlight some of the major findings regarding gaps in knowledge, major environmental problems of the country, and current and future activities to address them and to guide a transition toward sustainability. The lessons learned from this assessment can be applied to other megadiverse developing countries.

## The Mexican ecosystem assessment

Mexico's ecosystem assessment, published as the *Capital Natural de México* (CNM; CONABIO 2006, 2008a, 2008b, 2009, Sarukhán et al. 2010), was inspired by the Millennium Ecosystem Assessment conceptual framework but was adapted to the circumstances and characteristics of one of the most biologically and culturally diverse countries in the world. Its objective was to provide an organized account of the published knowledge (*sensu lato*, including literature, public databases, and public cartography) about the natural capital of Mexico, as well as of the status of conservation and sustainable use of its components, structure, and functioning. The work started in 2005, with a number of design meetings among the stakeholders. These included scientists, members of conservationist nongovernmental organizations, and government officers. The output of these meetings was an outline of the structure and contents of a five-volume work, accompanied by Web pages and databases. The inclusion of multiple stakeholders in the design phase was intended to achieve a balance among legitimacy (a participatory open process not influenced by politics), credibility (involvement of experts using traceable primary data), and relevance (providing information about specific problems) of the information analyzed and synthesized in the assessment (see Cash et al. 2003).

Over a period of 3 years, data were organized and made available to chapter writers. This included major analytical efforts, such as the estimation of areas of distribution of most terrestrial vertebrates (about 4000 species) that were used to run the software for a comprehensive set of gap analyses (CONABIO 2009). Most of the chapters were written by multiple authors, under the leadership of two to five leading authors. The chapters were peer reviewed and compiled in volumes, three of which (out of a planned five) are already available online (CONABIO 2014). Because much of the information about ecosystem structure and functioning needs to be updated regularly, the design of CNM included online products with frequent updates. Most of the species databases are also available online, as is the image bank (CONABIO 2014). The three available volumes (CONABIO 2008a, 2008b, 2009) refer to the state of knowledge of Mexican biodiversity (volume 1), the state of conservation and the causes and trends of change (volume 2), and the status of public policy and sustainability (volume 3). Two forthcoming volumes will address human and institutional capacities (volume 4) and future scenarios (volume 5). A summary highlighting strategic actions is already published (Sarukhán et al. 2012).

The CNM provides a major synthesis of the knowledge and management of biodiversity in Mexico. More than 700 scientists, government officers, and nongovernmental organization members participated. The key priority issues for future attention, as well as new research areas and options for the conservation and sustainable management of Mexico's threatened biodiversity were highlighted (Sarukhán et al. 2010). In the present work, we present a brief description of the major results of the CNM. It would be impossible and unnecessary to attempt an exhaustive description, and, therefore, we will simply provide some highlights and conclude with some general thoughts on the Mexican experience, lessons, and management actions that may be useful to other developing and megadiverse countries.

## Knowledge about the biodiversity of the country

The first volume of the CNM addresses the status of the knowledge (and the gaps therein) of the components, structure, and functioning of the biodiversity of Mexico, from genetic variability within species to ecosystem diversity, including a chapter on traditional indigenous knowledge (CONABIO 2008a).

The tradition of studying plants and animals in Mexico is centenarian. Not only is the knowledge of the hundreds of Mexican indigenous groups varied and deep, but the tradition of studying the botany and zoology of Mexican species started early in the colonial period. A growing amount of this knowledge is organized in the National Biodiversity Information System (SNIB), under the supervision of CONABIO. This information system comprises nearly 9 million georeferenced specimen records, housed in more than 1300 collections in Mexico and abroad, corresponding to about 90,000 names of species described for Mexico. This list was made available for the first time in the CNM, together with databases for endangered species, genetic resources, invasive species, and cartographic information. There are more than 4400 environmental, infrastructural, and socioeconomic geographical information system files, made available as shape files and as Web services relevant to biodiversity (CONABIO 2014). More recently, products from remote sensors, related to ecosystem conditions (e.g., fire and burnt area detection), are generated and made available on a daily basis. In addition, marine photosynthetic activity is reported regularly on CONABIO's Web site.

Despite the continued growth in primary data on biodiversity, important knowledge gaps remain. Some of the largest knowledge gaps in biodiversity relate to large and taxonomically complex groups with few (or no) specialists, including many invertebrates, fungi, algae, microorganisms, and marine life in general (MA 2005, CONABIO and UNDP 2009). SNIB holds records of less than 25% of the arthropods, crustaceans, and other invertebrate species known in Mexico, which, collectively, account for less than 6% of the total number of records. Filling the gap of knowledge for taxonomic groups such as nematodes; mites; and, even worse, mycorhizal and endophytic fungi, bacteria, and protozoa is a gargantuan task that will not be successfully tackled without major and long term-investments in human capital and institutions (Samper 2004) or major technological breakthroughs.

**Figure 1.**
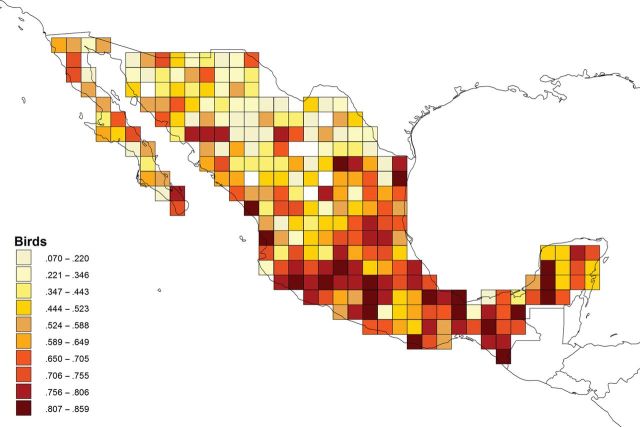
The completeness of knowledge index (KI) for the birds of Mexico (approximately 400,00 records of approximately 1000 species), at a resolution of 7100 square kilometers. A KI value of 1 means that no new species are added when new individuals are collected, whereas a KI value of 0 means that every new record is a new species. Source: Reprinted with permission from CONABIO (2008a).

Furthermore, the knowledge of species ranges is far from satisfactory, even in the case of terrestrial vertebrates, such as birds, one of the best represented groups in the SNIB (more than 75% of the possible total). As is shown in figure 1, increased sampling effort throughout the country continues to reveal the presence of species previously unrecorded from those sampling localities (CONABIO and UNDP 2009).

Fortunately, for these more conspicuous groups, engaging the public to collect the vast number of data required to better understand the large-scale patterns and temporal trends of change in biological diversity (Bonney et al. 2009) is a very viable strategy. The CNM highlighted the acute need for monitoring populations, at least in the major groups of vertebrates. This can be achieved by creating or supporting citizen science platforms that observe careful quality assurance and control procedures, along with local capacitation programs. For example, CONABIO's program aVerAves (CONABIO 2014), a version of eBird adapted for Mexico, has compiled over 1.5 million observations of 97% of the ornithofauna in Mexico, provided by over 3000 volunteers, which has considerably increased the SNIB data for birds.

Another fundamental challenge identified in the CNM relates to knowledge gaps for the sustainable management of ecosystems based on land use and cover change information. Currently, it is relatively easy to measure the status and basic properties of ecosystems (i.e., land use and land cover) using remote sensing at intermediate resolutions. To date, national-level land cover and land use maps produced by the National Institute of Statistics and Geography at scales of 1:250,000 have been used to assess deforestation trends, carbon dioxide emissions, and protected-area effectiveness (Cairns et al. 2000, Figueroa and Sánchez-Cordero 2008, CONABIO 2009, Kolb and Galicia 2012). However, discontinuity or long lags in time-series imagery (e.g., periods of 5 years or more), as well as inconsistent classification schemes and definitions between maps, seriously limit the usefulness of such information to correctly assess issues such as deforestation rates at adequate resolutions for multiple decisionmaking scales and to elicit prompt conservation responses (Mas et al. 2004).

Monitoring programs in which coarse spatial resolution satellite images are used are already in place to detect shorter-term land cover changes at the national scale, with the participation of various government institutions, including CONABIO (Colditz et al. 2012). At an intermediate-scale resolution (1:50,000) Conabio has implemented a nationwide ecosystem-monitoring program for mangrove areas with SPOT satellite images and high cartographic accuracy (greater than 90%; Rodríguez-Zúñiga et al. 2013).

Although ecosystem function metrics are much more difficult to quantify, CONABIO is currently building partnerships with several governmental and academic institutions and nongovernmental organizations, with the aim of developing satellite-based operational systems calibrated with *in situ* data to obtain, process, analyze, and distribute data on the health of and changes in key ecosystems, beginning with surface oceanographic processes. Such data are of particular importance to supporting both decisionmaking and the Marine Biodiversity Observation Network (Muller-Karger et al. 2013). The Satellite-Based Ocean Monitoring System in CONABIO provides information for the analysis of patterns in critical oceanographic processes, such as marine productivity, harmful algal blooms, and thermal stress in coral reefs (Cerdeira-Estrada and López-Saldaña 2011). Likewise, efforts are already in place to map the Mesoamerican reef benthic habitats with high-resolution satellite images, along with the the development of a monitoring network to measure coral temperature and bleaching, using a citizen-science platform (CONABIO 2014). The plans also involve creating a countrywide buoy network and the Satellite-Derived Bleaching Coral Reef Early Warning System (*SATcoral* in CONABIO 2014).

Although applied research on different aspects of genetic diversity is also a priority—in particular, to inform efforts of species conservation and recovery programs (e.g., the Mexican wolf, the California condor, the vaquita porpoise, the scarlet macaw) as well as conservation planning and sustainable use and restoration projects—only about 200 species in Mexico have been studied regarding their genetic diversity (CONABIO 2008a). In absolute terms, this is a small quantity, but few developing countries have this amount of information about the genetic structure of the populations of their species, which underscores the need to close this global knowledge gap. Of particular importance is to increase applied research for the management of genetic resources in the case of the cultivated plants that originated in Mexico (and other countries rich in agrobiodiversity), as well as their wild relatives (i.e., traditional management systems and *ex situ* conservation policies).

Another critical research area is that of environmental restoration, in which the large gaps remain disproportionate to the need of recovering degraded ecosystems. Among the main tasks ahead are to establish formal professional education programs, to consolidate research lines, and to promote the formation of highly qualified human resources, all of which should be considered priorities in current science policies. Knowledge gaps in reforestation programs include aspects as basic as the propagation of native species appropriate not only to restore ecological conditions but also to cover the uses by local people in a particular ecosystem or region (e.g., Suárez et al. 2011).

Research on community resource management has often proved that traditional and communal management practices could significantly contribute toward a model of sustainable development (e.g., Bray et al. 2005, Ellis and Porter-Bolland 2008). Consequently, rescuing, systematizing, and assessing the knowledge about the biodiversity of rural and indigenous communities in a culturally diverse country are particularly important tasks. Boege (in CONABIO 2009) presented a detailed account of indigenous conservation activities, and de Ávila Blomberg (in CONABIO 2008a) compiled an exhaustive list of zoological and botanical knowledge for nearly 60 indigenous groups.

## The conservation status of ecosystems and the threats to biodiversity and ecosystem services

As in other megadiverse countries, the exuberant biological diversity of Mexico has historically provided considerable benefits to the population of the country. However, such biological capital has been dramatically affected by a number of direct (i.e., proximate) and indirect (ultimate) drivers of biodiversity change, as is described in the second volume of the CNM (CONABIO 2009). The ultimate drivers, rooted in unsustainable economic growth and erroneous policies of exploitation of natural resources, have brought about a severe erosion of the country's biodiversity and of the environmental services crucial for human well-being.

Mexico's assessment revealed that the main proximate drivers of the deterioration of Mexico's ecosystems and the biodiversity that they hold has been land use and land cover change. Indeed, habitat destruction (i.e., conversion to other types of use) and fragmentation are recognized to have had major impacts on all of the terrestrial and aquatic ecosystems of Mexico. For instance, by 1976, the original coverage of terrestrial primary vegetation had been reduced to 54%, and by 2002, the country's major terrestrial ecosystems (tropical and temperate forests) only covered 38% of their original extent, with the greatest impact occurring in tropical systems. By 2011, the original extent of vegetation cover in primary and secondary condition was reduced to 72% (INEGI 2013). During the 1980s, when global ecosystems experienced a major pulse of deforestation, Mexico was losing approximate 600,000 hectares per year. Recently, the deforestation rates have declined in many regions of the country, but this is in part because the remaining areas are inaccessible, deemed no longer deforestable (e.g., they are viewed of low commercial value) or are under some regime of formal protection. Beyond this, a significant portion of the remaining vegetation is composed of small, isolated fragments or is represented by secondary growth (i.e., vegetation undergoing regeneration after being subjected to some degree of disturbance). In general terms, around 45% of Mexico's territory has been affected by soil degradation (CONABIO 2009).

Although less quantitatively documented, the degradation of marine and coastal ecosystems in Mexico has also been severe (CONABIO 2008a). For example, the healthy dynamics of coastal lagoons, which are one of Mexico's most crucially important ecosystems in terms of environmental services (Aburto-Oropeza et al. 2008), have been seriously affected on the Pacific coasts of Mexico by the damming of almost all rivers for hydroelectricity and agricultural irrigation. The sediments that, in the past, reached the coast and contributed to the buildup of sandbars, beaches, and coastal accretion, which, in turn, helped to rebuild the coast after storms, are now being trapped in the bodies of dammed lakes, making the coasts more vulnerable to the consequences of large-scale environmental change (López-Medellín et al. 2011).

Beyond habitat destruction, the overexploitation of resources (logging, hunting, overfishing, illegal trading of biological resources of plant and animal origin) and exotic invasive species have affected the biodiversity of the country. Going forward, these factors, together with climate change, are likely to continue the deterioration of ecosystems unless effective action is taken immediately. In this context, a critical research agenda is the understanding of the interactive effects of these drivers, which will most likely reinforce themselves and which have the potential to generate tipping points (Barnosky et al. 2014), with potential irreversible changes in many regions and ecosystems of the country.

The situation described above has affected the biodiversity of the country in terms of the extinction of species (a total of 127 plants and animals) and the number of threatened species, 2493 of which are currently under the Mexican Official Standard (NOM-059-SEMARNAT-2010). Biological extinction, however, is not restricted to the national or global loss of species but is also critically reflected in the loss of local populations of many species, even when other populations of such species may still be present in other regions or countries. Most unfortunately, we lack reliable estimates of population extinction, but, given the known rates of habitat loss, we can infer that this aspect of biodiversity loss must be of considerable magnitude in Mexico (for details, see CONABIO 2009). The loss of local populations, as well as the decline in abundance in many populations, represents a critical conservation aspect, because it is at the local level that the ecological services provided by ecosystems and biodiversity acquire special significance for local communities. Our assessment documents that provisioning services such as food production derived from agriculture, cattle production, fisheries, and aquaculture have been used in unsustainable ways, which has led to their deterioration in various degrees and which represents a major challenge for biodiversity conservation going forward, particularly in light of the future demand of food by the growing population. It is crucial—and, indeed, a matter of national and international security—that society engage in a discussion of how to develop a program of sustainable food production without further deteriorating the natural capital and, to the extent that is possible, promoting the restoration of degraded areas in which unsustainable productive activities have been conducted. Indeed, the productive activities of forestry, cattle production, and fisheries and the recollection of natural products need to be conducted under diversified-use mosaic landscapes with management schemes that integrate the use of multiple species; that are associated with the maintenance of multiple ecosystem services; and that engage local cultures, respecting their traditional knowledge regarding the use of resources and their cultural values.

The CNM also reviewed traditional conservation actions—specifically, protected areas and *in situ* and *ex situ* conservation methods and conservation in indigenous lands. Clearly, one of the most consolidated institutional tools for addressing the problems of biodiversity loss and for promoting its conservation is the establishment of natural protected areas. Collectively, the protected areas of Mexico encompass 10% of the land surface, 23% of territorial seas, 12% of the continental shelf, and 1.5% of the exclusive economic marine zone (CONANP 2014). The assessment provides a comprehensive analysis of the distribution of ecosystem types across the different protected areas, the concentration of species and endemic taxa, their conservation status, and the land tenure systems associated with them. Two results of this analysis standout: On one hand, it is clear that their coverage has the potential of protecting only a limited contingent of the entire biodiversity of the country (CONABIO 2009). Moreover, inadequacies in natural protected area management, financial limitations, and gaps in the representation of biodiversity remain as constraints for effective biodiversity protection and a crucial conservation agenda in Mexico (Bezaury-Creel 2009, CONABIO 2009). On the other hand, this implies that the fate of a considerable proportion of the country's biodiversity will depend on how the remaining 90% or so of the territory, with human communities present, is managed. Therefore, conservation actions will have to be conducted in mosaic landscapes with different land uses and involving local human communities, in contrast to the traditional scheme of parks with no people, a situation applicable to other biodiversity-rich countries (Sarukhán and Dirzo 2012). We will come back to this point below.

## Other efforts to reverse trends in biodiversity loss

The results of the first two volumes of the CNM paint a stark landscape. Efforts to preserve biodiversity have a long history in Mexico (González and Sánchez 1961, CONABIO 2008b). Nonetheless, the prevailing attitude in governmental circles, particularly during the second half of the twentieth century, was that economic development could be based on the intense use of seemingly unlimited natural resources, with priority given to sectorial interests over public social welfare and a healthy environment. It is not an exaggeration to say that it was not until 1994, with the creation of the then Secretariat of Environment and Natural Resources and Fisheries (Semarnap, renamed Semarnat in 2001, when the fisheries sector was integrated into the Ministry of Agriculture), that more-comprehensive conservation and resource management approaches started to consolidate. In volume 3 of the CNM (CONABIO 2008b), an analysis is presented of less conventional approaches to biodiversity conservation that respond to the reality of a high beta-diversity country (CONABIO 2008a) with a large (approximately 30%) peasant population. This combination converges with the implication mentioned above that a strategy of conservation based only on traditional protected areas, in which people are excluded or restrained in their activities, will conflict with the widespread and ancient human occupation of the territory. Alternatives exist—namely, sustainable forestry, sustainable use of wildlife, ecological restoration, payment for ecological services, and ecological planning of the territory. These forward-thinking alternatives have been bolstered by new laws and regulations regarding the use and conservation of biodiversity. For example, the National Forestry Commission (Conafor) has implemented a novel economic instrument to strengthen conservation efforts through the National Programs of Payments for Hydrological Services (PSAH) and for Biodiversity Services (PSAB), with new strategies to evolve from being a merely subsidiary scheme (that can hardly compete with land opportunity costs or that cannot guarantee the long-term protection of ecosystems in properties with PSAH or PSAB contracts), through the promotion of local markets of ecosystem services through matching funds to support institutional arrangements, and through the creation of a long-term funding program for the conservation of forest ecosystems that harbor globally important biodiversity elements (León et al. 2012, CONAFOR 2014).

The promotion of biodiversity-friendly but productive activities should be a priority of multiple stakeholders (CONABIO 2008b). One of the best examples is the sustainable community management of diverse forest ecosystems. Among others (see, e.g., Bray et al. 2005), a good instance occurs in Ixtlan de Juárez, Oaxaca, where the community has engaged in a forestry management program, certified internationally as sustainable and with a very high degree of integration, and which has culminated in the design and production of furniture that constitutes a profitable local industry with significant economic revenues that directly benefit the community (Carabias et al. 2010).

The sustainable use of wildlife species, either for game or fisheries, is becoming more common. Many communities and smallholders have achieved positive ecological and economic impacts through the legal use of biodiversity in wildlife management units—in particular, in the north of the country. In fisheries, one example from marine ecosystems is that of the Pacific red lobster (*Panilurus interruptus*), certified since 2004 by the Marine Stewardship Council in Baja California (Carabias et al. 2010) and managed by a cooperative that operates as a social enterprise. It reports sustained annual catches of 1600 tons of product for the last 10 years or so.

There is a growing number of examples like these already operating in Mexico (Carabias et al. 2010, Porter-Bolland et al. 2013). Undoubtedly, these initiatives represent a foundation of environmental sustainability and social welfare in rural areas in the long term (e.g., Bray and Velázquez 2009).

Without a doubt, the secure and sustainable production of food to satisfy future national requirements is an issue of national security. How to achieve food security in a sustainable and biodiversity-friendly way is a challenge of historical proportions (Foley et al. 2011). A strategy identified in the CNM is to promote the process of diversification and the productive reconversion of agriculture and cattle ranching to multifunctional, agroecological, or agroforestry systems of production (CONABIO 2008b), many of which can be developed for particular environmental conditions by assessing and rescuing traditional or low-input management practices. The worldwide empirical evidence supports the idea that peasant and small-scale family farm operations adopting agroecological methods can be can be more sustainable and, under the right conditions, as productive as conventional agriculture (Perfecto and Vandermeer 2010)—in particular, when environmental degradation and pest outbreaks due to agroindustrial practices are accounted for (González 2012).

Finally, Cervantes and colleagues (in CONABIO 2008b) showed that, although *in situ* conservation efforts will always be more cost effective than *ex situ* or restoration strategies, an imperative task to be accomplished in Mexico is the development and implementation of a national ecosystem restoration policy that corresponds to the magnitude and degree of ecosystem degradation (CONABIO 2008b). The promotion of reforestation and soil improvement activities have been in place since the beginning of the twentieth century; however, it was not until the 1990s that a more integrated—but limited—approach to restoration was incorporated into government programs and instruments, whereas the restoration efforts conducted by nongovernmental organizations and universities have also increased in recent decades (Carabias et al. 2007, Lindig-Cisneros 2010). One of the main challenges that the country is facing is to effectively integrate ecological and traditional knowledge, technical capacities, and the preponderant role of social issues in all restoration programs in a way that engages the active and long-term participation of landowners, as well as increases financial and governmental support. For instance, most programs only encourage participation through economic incentives, without considering that nonfinancial interest can play an important role (Carabias et al. 2007, Cotler et al. 2013).

## Conclusions

**Figure 2.**
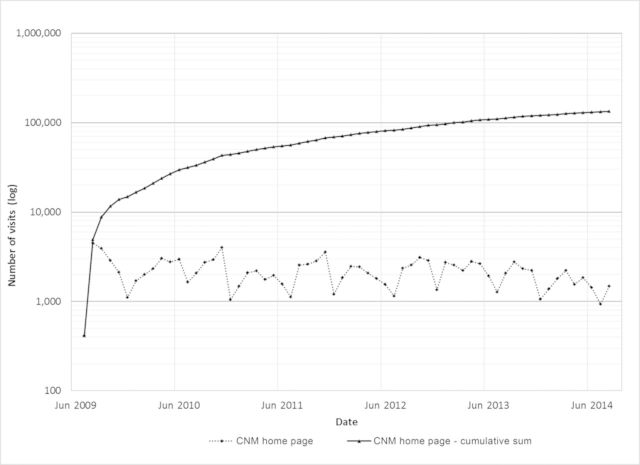
Accumulated (solid line) and number of accesses to the Capital Natural de México (CNM) Web site (July 2009–August 2014). Source: The data are from the Google Analytics report of CONABIO (2014).

The effort to organize and compile the multivolume CNM, with its associated online support information was very significant, and the results have been well received. In addition to the broadly distributed 3000 printed volumes, the CNM volumes have been widely consulted since they were made available on CONABIO's Web site: The CNM part of the Web site has been visited 133,718 times, with an average of 2156 visits per month (figure 2).

Both the product, itself, and the process leading to it provided several important lessons that may be useful outside of Mexico. Below, we first derive the positive lessons and, later, some of the knowledge gaps, problems, and improvements needed for future assessments that address the science–policy gap.

### Positive lessons

First of all, without the accumulated basis of knowledge that Mexico has available, much of the results of the CNM would have remained speculative at best. Since colonial times but increasingly after the revolution, the country has created a substantial base of information about its biodiversity (e.g., books, published papers, scientific specimens, digital cartography, remote-sensing images; see Soberón et al. 2010). With the creation of CONABIO in 1992, a sustained effort to convert such information to machine-readable formats and to make it available to the public via the Internet has created a formidable tool for analysis and synthesis (CONABIO 2012). This tool was fully used for the CNM effort, and it is difficult to see how the depth and broad scope of the CNM could have been achieved without it. Because several megadiverse and developing countries have biodiversity information agencies or institutions in place (Sarukhán and Dirzo 2012, Soberón 2014), they are in a position to use their resources for an effort similar to Mexico's.

Second, as was stated earlier, without the participation of a high number of stakeholders, the CNM would not have had legitimacy, relevance, or credibility (Cash et al. 2003). In fact, the whole exercise, in a way, came to exist because of the demand of some of the major nongovernmental organizations working in Mexico for the establishment of conservation priorities, given the steady rate of ecosystem deterioration that the country experiences. The participation, from the design phase, of both international and local conservationist nongovernmental organizations was essential for the good reception of the work. However, a very large number (more than 700) of Mexican and foreign scientists acted as coordinators, writers, or reviewers. This is an unprecedented effort and provides the work with an authority that could not have been obtained otherwise. From the beginning, members of several federal agencies were also involved in the CNM. The project had the support of the minister of the environment, and with the direct support and participation of the heads of the National Institute of Ecology and Climate Change, the National Commission of Protected Areas, CONAFOR, and CONABIO. We believe that this multistakeholder participation will ensure that the CNM will remain accepted and used for many years.

Third, although the CNM contains a comprehensive analysis of the status of and gaps in conventional tools for conservation (protected areas in their different versions, *in situ* and *ex situ* protection of threatened species), one salient point is the variety of unconventional approaches taking place in Mexico, including sustainable forestry, sustainable and biodiversity-friendly coffee, ecotourism, sustainable management of wildlife species, and sustainable fisheries practice. All of these, incipient as they may be, show the way toward a way of management of the landscape that is compatible with economic activities but that does not substantially degrade biodiversity or ecosystem services. Mexico is privileged in that regard, because the heritage of agricultural and environmental knowledge spans millennia (García-Barrios 2009), and many grass-roots organizations have shown that there are options to harmonize the conservation, restoration, and sustainable management of biological diversity, with tangible economic benefits for the Mexican population, especially those whose livelihood depends on ecosystems.

### Problems

Substantial as the knowledge of biodiversity of Mexico already is, the CNM revealed the existence of serious gaps. Among the main ones is the lack of knowledge about minor or inconspicuous groups. This is obvious for invertebrates, and for some groups, like the fungi, nematodes, acari, and bacteria, the documentation work is still in a pioneering stage. Knowledge about the structure and functioning of biodiversity components is woefully insufficient. The information about the interactions among species (e.g., pollinators, seed dispersers, herbivores, symbiotic mutualists, diseases, keystone species, trophic web structures, among a long list) is limited to a handful of species and sites. The ecosystem processes that cannot be studied using remote sensing remain basically unmeasured, bar a handful of studies. Long time series of the numbers of species, vegetation structure, or changes in genetic composition are almost nonexistent. With the exception of species monitoring, these problems can be solved only by a sustained investment by the government on the scientific apparatus of the country (Martínez et al. 2006). However, the monitoring of certain taxonomic groups can be achieved by an increased participation of society in the recording of attractive species and processes (e.g., migrations, phenology of flowering plants), and this is already happening in Mexico (Sarukhán et al. 2012).

Although the CNM project included scientists, federal government officers, and large nongovernmental organizations, several major stakeholders did not participate. This is very much an issue of scale. At a state and—even more—at local levels, the number of actors multiplies, their concerns change and diversify, and the available high-resolution data indispensable to address local-level questions are mostly nonexistent. Moving from a national level to more local levels will require substantial resources (the cost of the CNM was about US$500,000) and will be much more complicated to organize. Eventually, these will be required, and, to an extent, some attempts have already been made by local nongovernmental organizations and by local universities or governments (e.g., the State Biodiversity Strategies promoted by CONABIO). However nothing in the proportional scale of the CNM has been attempted at subnational levels in Mexico. The sheer difficulty of changing scales should be a sobering reflection to recent global organizations that, at least in their discourse, have the objective of advising governments at a local level (IPBES 2012).

The CNM represented an unprecedented work of data systematization, reflection, and analysis. Although, as we have shown, thousands are accessing the books and databases, we believe that the most important result of the CNM is actually the experience of an organized, multistakeholder work that took place over many years. It is becoming more common for teams of scientists, nongovernmental organizations, and government officers at the federal level to tackle together complicated environmental problems, such as the monitoring of the results of the REDD+ program (REDD+ 2014) or the management of protected areas. This is a very positive development, which was taken to a new level in Mexico through the experience of the CNM. Whether this will serve to change the environmental degradation trends that Mexico continues to experience depends on the engagement of policymakers and the support of society at large.
